# Selecting interventions to promote fruit and vegetable consumption: from policy to action, a planning framework case study in Western Australia

**DOI:** 10.1186/1743-8462-5-27

**Published:** 2008-12-24

**Authors:** Christina M Pollard, Janette M Lewis, Colin W Binns

**Affiliations:** 1 Curtin University of Technology, GPO Box U1987 Perth WA 6845, Western Australia, Australia; 2 North Central Metropolitan Primary Care Partnership, PO Box 1681, Preston South VIC 3072, Victoria, Australia

## Abstract

**Background:**

The Department of Health in Western Australia identified access to, and daily consumption of recommended amounts of fruit and vegetables, as priority health determinants. The numerous factors that influence supply and consumption of fruit and vegetables indicated that a comprehensive approach would be required.

A government and non-government sector steering group was set up to select priority interventions using the National Public Health Partnership's *Framework for Implementing Public Health Strategies*. This structured framework was used for developing strategies to improve fruit and vegetable consumption and supply, and to identify implementation priorities.

After one year a desktop audit of progress on framework interventions was undertaken.

**Results:**

The structured framework led to a plan for defined actions, partners, costs, and performance indicators for strategies to improve fruit and vegetable consumption and supply. Lead agency custodians for management of the selected interventions were identified.

After one year there was significant progress in the implementation of a number of the high-ranking interventions. The exception was interventions that provide the infrastructure support such as research and development capacity, information systems.

**Conclusion:**

A structured framework and stakeholder participation assisted in developing a fruit and vegetable implementation strategy. Engagement and commitment of influential and diverse stakeholders is needed, not just for program support, but particularly in the areas of food and nutrition policy development and providing the infrastructure support required. Further work is required to develop performance outcomes and cost effectiveness measures for many of the strategies that have been proposed to address portfolio objectives.

## Background

The impact of government policies in relation to the provision of a healthy diet available to all (nutritional quality), needs to be assessed in relation to overweight and obesity [1]. The highest rates of obesity occur in the populations with the lowest socioeconomic status [2]. Poverty, deprivation and limited access to healthy foods explains some of the differential. Calories provided by whole grains, fresh produce and lean meats have been found to be more expensive than those from refined grains added sugars and added fats [3]. Although fruit and vegetables are an expensive source of dietary energy, they provide key nutrients at reasonable costs [4]. In contrast, although refined grain foods, added sugar and hardened fats are usually affordable, enjoyable and readily accessible, they contain the least nutrients per unit cost [4].

### Increasing fruit and vegetable consumption as an obesity prevention strategy

Emerging evidence suggests that increasing fruit and vegetables may assist dietary weight management strategies to prevent obesity [5,6]. Energy density is reduced by higher intake of fruit and vegetables [7]. Eating larger amounts of fruits and vegetables increases the feeling of satiety and results in the displacement of more energy dense foods [6]. People tend to eat a consistent daily volume of food, regardless of the energy content of the food, therefore, the energy density of food has an impact on the daily energy intake [3]. Incorporating more fruit and vegetables can reduce the overall energy density of the diet, promote satiety and decrease the total energy intake and increase diet quality [3,8,9].

Increasing fruit and vegetable consumption has been identified as a global public health nutrition priority [10,11] and has been the focus of population health in Australia [11-17]. Epidemiological studies estimate that substantial reductions in diet-related disease and health care costs would be achieved if fruit and vegetable consumption were raised to recommended levels [5,18].

### Addressing the influences on consumption of fruit and vegetables

Numerous factors that influence consumption of fruit and vegetables indicate that a comprehensive approach is required [19]. Consumption is influenced by structural factors impacting on supply [20]. Cost components of accessibility occur throughout the value chain: cost of production, processing, transport, wholesale and retail marketing practices [21]. Changes in any of these cost components can have an impact on either final price or cost effectiveness of selling in some locations, particularly rural and remote areas [22]. Alongside these supply factors lie individual eating behaviours [23,24].

Interventions need to address sociocultural, economic, educational and technical challenges to expand and address the fruit and vegetable supply chain. Strategies required include increasing:

• consumer awareness of benefits of healthy eating, motivation, and skills to increase consumption

• fruit and vegetable production and availability

• understanding of the components of consumer food choice attributes (taste, texture, form, price, convenience, quality and safety)

• innovation and the development of fruit and vegetable based food products

• opportunities for consumption in various settings, for example worksites

• implementation and evaluation of educational campaigns integrated with efforts to increase availability of and access to fruit and vegetables [25].

Government policy [14] and dietary guidelines [26-28] form the basis for most nutrition interventions in Australia. A planning process is required to select effective interventions and actions to bring about the desired changes. A planning framework was developed for the National Public Health Partnership to assist Australian government health departments with their planning, management, quality assurance and provision of public health interventions [29]. Barraclough & Gardner assert that, in Australia, if policy goals are shared between the Federal, State and Territory levels, the implication is that they will be pursued more vigorously [30]. Commencing in 2001, a systematic approach to planning the provision of public health interventions was used by the Department of Health Western Australia (DHWA) to prepare the "Eat Well WA-Fruit and Vegetable Strategy".

The Western Australian (WA) government had sponsored a social marketing campaign approach to increase fruit and vegetable consumption for over a decade, partnering with government and non-government sectors [31]. The success of partnerships between health and industry sectors working on the government's campaign depended on the right mix of organisational commitment, leadership, relationships, opportunities and capacity to achieve [32].

Although WA had the highest daily intake of fruit in 1995, and the second highest of vegetables of any other state or territory in Australia, levels are still lower than recommended for optimal health [33]. Consumer research identified the following barriers to increasing fruit and vegetable consumption in Western Australia:

• personal and family eating habits that are difficult to change

• false impression of already eating enough

• perceived high cost, particularly of fruit

• inconsistencies in price of fruit and vegetables

• lack of skills in preparation of tasty and convenient fruit and vegetable dishes

• perception that vegetables are time consuming to prepare

• concerns about pesticide residues and genetically modified foods

• lack of, or limited supply, poor quality and high cost (in isolated areas) [32,34].

## Method

The Department of Health in WA led the development of the "Eat Well WA-Fruit and Vegetable Strategy" (EWWAFV) to advise on and coordinate activities to improve access and increase consumption of fruit and vegetables. A framework with a systematic approach to planning the provision of public health interventions was used [29]. The approach ensured that a comprehensive range of intervention types were considered and the capacity to carry out the interventions. The following steps were used:

1. Identify the determinants of health

2. Assess the risks and benefits posed by each determinant to identify what should be addressed

3. Identify intervention options and appraise them

4. Decide on the portfolio of interventions

5. Implement the portfolio

6. Evaluate the portfolio [29].

Literature and program reviews identified health determinants and their risk/benefits. Based on these, two health determinants were chosen:

• access to fruit and vegetables

• consumption of at least 300 g (2 servings) fruit and 375 g vegetable (5 servings) daily [35].

A comprehensive approach to intervention planning covers policy, program interventions, and the infrastructure required to support them. The full range of intervention types were considered to decide appropriate actions. Types included public policy development, legislation and regulation, resource allocation, engineering and technical interventions, incentives (financial and other), service development and delivery, education (including skills development), communication (including social marketing), collaboration/partnership building (community and intersectoral), community and organisational development (including organisational policy) [29]. The Department of Health outlined the types of interventions that could be considered using available evidence and the intervention recommendations from a national case study [16,17]. Next, stakeholders were consulted to capture other possible interventions. Involvement of both the government and non-government sector would be crucial for effective action in implementing strategies [36].

Two distinct management portfolios were defined, which related to access and consumption of fruit and vegetables. These recognised that better access to fruit and vegetables is likely to assist in increasing consumption, while increasing consumption has implications for improving access. Portfolio one addressed the access to fruit and vegetables health determinant with the management objective "to increase and sustain access to high quality, safe, affordable vegetables and fruit". Portfolio two addressed the health determinant relating to consumption with the objective "to increase consumption of fruit and vegetables by people in WA to meet or exceed recommendations".

A steering group with representatives from relevant sectors was formed to lead the development of the strategy and select priorities for action. Members represented the retail, hospitality, catering, education, horticulture, agricultural and transport sectors, environmental health, regional health, produce retailers, fruit growers, vegetable industry, non-government health organisations, and consumers. Members were asked to weight the relative importance of interventions using the available evidence and their knowledge and professional judgement. The group assigned a score out of 10 to each intervention for its expected performance against specific criteria including: effectiveness, equity, feasibility, acceptability, timing and sustainability.

Lead agency custodians for management of the selected interventions were identified. Custodians were expected to be responsible for the overall management and communication between partnering organisations to implement the particular intervention. Partners required to support the implementation were enlisted. In addition specific actions, costs, and performance indicators or measures were identified and confirmed by the steering group. Key stakeholders from government departments, non-government organisations and the fruit and vegetable industry were invited to form a Steering Committee and an EWWAFV strategy workshop was held to initiate the process. Participants from relevant sectors were invited – government (health, agriculture, education, industry, training and transport), non-government health agencies, the fruit industry (from gate to plate), Foodbank WA, hospitality, and training, and horticulture industries. Attendees were asked to nominate to be part of a committee to oversee the development of a ten-year strategy and implementation plan. The committee reviewed interventions in the two management portfolios, their suggestions were added and priorities for action determined.

## Results

The strategy development process took over a year with meetings every four to six weeks. The stakeholders engaged extensively in the strategy development and intervention assessment process. Most of the group attended meetings and were active throughout the process. Tables 1 and 2 show the 33 high-ranking interventions in both portfolios in 2002. A mass media campaign to promote fruit and vegetables was identified as a priority activity with the Department of Health as the lead agency due to their experience in and commitment to social marketing campaigns. Research and development were priority activities for identified industry groups. The hospitality industry (including hospitality training organisations), the Departments of Agriculture, Transport, and Environmental Health were custodians for the management of a number of fruit and vegetable supply strategies. Individual industry grower groups were identified to promote specific fruit and vegetables as a priority activity.

**Table 1:** High-ranking interventions in Portfolio 1 Goal: Health Determinant: access to fruit and vegetables (f&v), assessment of actions at one year (∥ = not started, ▶ = some progress, ▶▶ = significant progress). Management objective: to increase and sustain access to high quality, safe affordable fruit and vegetables

***Policy interventions***
*Public Policy development*
1. Develop and support f&v consumption guidelines and position statements-∥
2. Develop and support nutrition policies promoting f&v in schools-▶▶
*Legislation and regulation*
3. Advocate for legislation for restriction of food advertising directed at children-▶
*Community & organisational development (including organisational policy)*
4. Support local initiatives and organisations to develop and implement food and nutrition policies and improve access to f&v-∥
5. Encourage the development of retailer training policies for handling f&v e.g. 'Retailer of the year' award-▶
**Program interventions**
*Incentives (financial and non-financial)*
6. Support award schemes that increase access to f&v for consumers:-▶▶
- Worksite
- Childcare & Schools (e.g. STARCAP^1 ^and Start Right-Eat Right^2^)
- Hospitality (e.g. Gold Plate Award^3^)
- Supplier/retailer and transport operator accreditation/incentive schemes
- Product awards (e.g. WA Nutrition Awards^4^)
*Service Development and delivery*
7. Support the promotion of f&v in hospitality and catering training-▶
8. Support ongoing systems and food safety training e.g. HACCP^5^, SQF^6 ^and approved supply programs-▶▶
*Communication (including social marketing)*
9. Support dissemination of FSANZ policies and promotional materials relating to f&v-▶
10. Provide consumers with promotional materials on best conditions for storing fresh foods through retail outlets-▶▶
*Collaboration/partnerships(community and intersectoral*
**11**. Support welfare agencies in the provision of f&v (e.g. FoodBank^3^)-▶▶

**Infrastructure support**
*Identification and surveillance of determinants*
12. Collect and analyse information to assess f&v supply, cost, quality, access, sales/marketing (e.g. Market basket survey)-∥
*Information systems*
13. Develop systems to improve communication of f&v marketing & information through the supply chain eg price, quality, volume statistics-∥
*Research and development capacity*
14. Identify f&v supply issues in rural and regional development plans and make recommendations for action-∥
15. Identify and test assumptions about critical factors impacting on price, quality and access to f&v – remote, rural and urban-∥
*Plant and equipment*
16. Advocate for appropriate f&v storage facilities in remote community stores-∥
*Leadership*
17. Establishment of a Western Australian Taskforce on Equity in Food Access-∥

^1 ^Star Canteen Accreditation Program , ^2 ^[45], ^3 ^Foodbank Western Australia , ^4 ^WA Nutrition Awards , ^5 ^HACCP Based Food Safety Programmes and Endorsements , ^6 ^Safe Quality Food Institute

**Table 2:** High-ranking interventions in Portfolio 2 Goal: Health Determinant: consumption of 375 g vegetables (5 servings) and 300 g (2 servings) fruit daily (f&v), assessment of actions at one year (∥ = not started, ▶ = some progress, ▶▶ = significant progress). Management objective: to increase consumption of fruit and vegetables by people in Western Australia to meet or exceed recommendations

**Policy interventions**
*Public Policy development*
18. Encourage whole of government organisations' policies to support f&v consumption-∥
*Resources allocation*
19. Support DOH resources allocated to support f&v mass media campaign: TV, radio, press, publications, point of sale, school activities, sponsorships, retailer and food service promotions-▶▶
*Community & organisational development (including organisational policy)*
Encourage the development of school/childcare food and nutrition policies and canteen foodservice guidelines e.g. STARCAP^1^, Start right – Eat Right^2^-▶▶
**Program interventions**
20. Increase the emphasis on the nutrition component in teacher training-∥
21. Address barriers to conducting school visits to f&v production sites and markets-∥
*Communication (including social marketing)*
22. Support statewide f&v campaigns consistent with Australian dietary guidelines recommendations e.g. retailer point-of-sale promotions, co-promotions/branding (e.g. Go for 2&5^®4^) – ▶▶
23. Increase public awareness of individual's health and economic benefits of eating more f&v (e.g. FOODcents^5^)-▶▶
*Collaboration/partnership building (community and intersectoral)*
Promote f&v tastings and demonstrations-▶▶

**Infrastructure support**
*Identification and surveillance of determinants*
24. Collect and analyse information to assess consumption and consumer attitudes to f&v-▶▶
*Information systems*
25. Disseminate research (e.g. publish food consumption and trends)-▶▶
*Research and development capacity*
26. Research best practice programs to increase f&v consumption-▶▶
27. Release f&v guidelines-▶▶

^1 ^Star Canteen Accreditation Program , ^2 ^[45]^3 ^[46]^4^[34]^5^[47]

A desktop audit of progress on framework interventions one year after the portfolios were developed revealed significant progress in the implementation of a number of the high-ranking interventions, see indicators of progress of actions on Tables 1 and 2. None of the infrastructure support interventions for Portfolio 1, access to fruit and vegetables, were started at one year, see Table 1.

## Discussion

Government response to health determinants requires a multi-strategy approach. Stirling et al (2007) developed multi-criteria mapping to obtain stakeholder assessments of obesity policy options [37]. In WA, to improve consumption and access to fruit and vegetables, government and non-government stakeholders were involved in the development of a statewide strategy. Key stakeholder judgements were required to move the list of interventions from what we 'could' do, to what we 'should' do [38]. During this time there was considerable sharing of information and insights. The judging and discussing of specified criteria lead to a cross sector learning process that enhanced the group understanding of the detail and scope of the strategies and enabled them to be assessed and ranked accordingly. The process enabled issues of ownership and cost to be considered by those accountable for the outcomes.

A limitation of the process is that people work in the context of what they perceive is possible and may underestimate the amenability for change. For example, at the time of the intervention selection the group thought that mandating guidelines for foods provided in school canteens was not a politically favourable option. Since then, governments in Australia have started to mandate for schools to provide food and beverage choices consistent with the Australian Guide to Healthy Eating; NSW initiated the process [39]. The WA school canteen accreditation scheme (STARCAP) intervention has provided a comprehensive infrastructure to support mandated policy directives.

It has been suggested that interventions be designed and delivered in ways that strengthen and support each other, for example, using social marketing campaigns like the Go for 2&5^® ^campaign to provide support, process and rationale for school canteen interventions [39]. An advantage of the process outlined here is that interventions that support each other can be identified and partnerships developed during the planning process.

Using this planning framework to develop management portfolios and assign responsibilities highlighted the need to also identify management performance measures including economic evaluation. Developing models to measure cost effectiveness for public health strategies that are implemented in a range of settings by practitioners from different backgrounds is a task yet to be undertaken. A review of the NSW state policy for child obesity prevention notes the importance of specific outcomes or set evaluation measures for actions addressing government policy [40].

It is important to note that there were some resources available for interventions at the time of selection. A reasonably well-funded government Health Promotion Directorate led the process. The Directorate had a 20 year history implementing social marketing campaigns and the Western Australian Health Promotion Foundation, Healthway, provided funds for community based health promotion interventions. Some actions identified to provide the infrastructure support for improving access to fruit and vegetables had not started at the time of the audit. The lack of action was not due to rethinking or abandoning the priority, it appeared to be due to the length of time required to instigate the more costly and complex strategies which required aligned action across sectors and engagement of external partners, such a research and development capacity and building information systems.

In 2003, the Government section responsible for leading the development of the portfolio was disbanded and the emphasis shifted from risk factor focus, for example nutrition, to a chronic disease prevention focus. Planning frameworks with designated custodians from a variety of sectors may enable work to continue during these types of organisational reforms.

Health promotion to improve nutrition will need to address wider food supply issues, particularly who controls and influences the food chain and thus individual and community food choices [41]. The global food supply is also an issue of local concern [42]. Influential and diverse stakeholders will need to be engaged in and committed to the process, particularly in the areas of food and nutrition policy development and providing the infrastructure support required.

Further work is required to develop performance outcomes and cost effectiveness measures for many of the strategies that have been proposed to address objectives.

## Conclusion

Comprehensive multi-component strategies that are implemented strategically over sustained periods of time are required to increase fruit and vegetable consumption [43,44]. Developing a State-based fruit and vegetable strategy using a defined framework for intervention highlighted the importance of stakeholder participation in the decision making process.

## Abbreviations

EWWAFV: Eat Well WA-Fruit and Vegetable Strategy; WA: Western Australia; DHWA: Department of Health Western Australia; HAL: Horticulture Australia Limited.

## Competing interests

J Lewis and C Pollard worked for the Department of Health (DHWA). Horticulture Australia Limited funded Curtin University of Technology to assist with the preparation of publications.
